# Treatment of Relapsed Acute Lymphocytic Leukemia in Adult Patients

**DOI:** 10.1007/s11864-024-01213-4

**Published:** 2024-06-25

**Authors:** John C. Molina, Hetty E. Carraway

**Affiliations:** Leukemia Program, Taussig Cancer Institute, Cleveland Clinic, 9500 Euclid Avenue, Cleveland, OH 44195 USA

**Keywords:** Acute lymphoblastic leukemia, Blinatumomab, Inotuzumab, CAR-T

## Abstract

For adult patients diagnosed with relapsed B cell-ALL (B-ALL), there have been significant improvements in available treatment options following the FDA approval of novel cellular and immunotherapy approaches – blinatumomab, chimeric antigen receptor (CAR) T therapy, and inotuzumab. For the last several years, research has focused on gaining a better understanding of the effects of specific disease and patient characteristics on long-term outcomes with each of the FDA-approved agents. In combination with the better prevention and management of unique, treatment-specific toxicities, providers can now select the best available treatment option for each individual patient diagnosed with relapsed, adult B-ALL needing therapy. This has allowed more patients to proceed to consolidative hematopoietic stem cell transplant (HSCT), and long-term data has even brought into question the need for HSCT for long-term durable remission for all patients. However, with the adoption of blinatumomab, CAR T therapy, and inotuzumab in front-line treatment regimens, it remains unclear what effects this will have on patients with relapsed B-ALL following exposure to these novel cellular and immunotherapy therapies. Unlike B-ALL, similar advances have unfortunately not yet been realized in T cell-ALL (T-ALL). Currently, new therapeutic approaches are underway to utilize similar targeting strategies that have been successful in B-ALL – monoclonal antibodies, bispecific T-cell engagers (BiTE), and CAR T therapy. Like B-ALL, the only existing approved therapy for relapsed T-ALL, nelarabine, is now used in the upfront treatment setting potentially limiting its utility in relapsed disease. Over the next several years, the hope is for patients diagnosed with T-ALL to experience the drastic improvement in outcomes as has been seen for patients diagnosed with B-ALL over the last decade.

## Introduction

Over half (~ 3,000 cases) of acute lymphoblastic leukemia (ALL) diagnoses occur in patients ≥ 18 years old and ALL accounts for ~ 20% of all adult leukemia diagnoses. Each year, there are approximately 6,000 new cases of B and T-cell ALL in the United States causing an estimated 1,580 deaths annually [1].

For the last two decades, the 5-year overall survival (OS) for ALL has steadily improved across all age demographics with the largest gains in children 1–14 years of age who now achieve a > 93% 5-year OS following upfront intensive therapy. In contrast, the 5-year OS for adults 20–39 years of age is only 59% with further decrements in overall survival as patients age; with 43% 5-year OS in patients 40–59 years old, 29% in patients 60–69 years old, and only 13% in patients 70 years or older [2]. Despite initial complete response (CR) rates of ≥ 90%, over half of adult patients diagnosed with ALL will ultimately relapse with a median post-relapse OS of 4.5–6 months and a 5-year OS of less than 10% [1].

A major barrier for patients with relapsed ALL has been the limited utility of chemotherapy-based salvage regimens. Due to the low remission rates and short duration of the remissions achieved with salvage chemotherapy, only 10–30% of adult patients were previously able to proceed to a curative, allogeneic hematopoietic stem cell transplant (HSCT), irrespective of age [3, 4].

The limited efficacy of salvage chemotherapy drove the development of novel treatment approaches to try to improve long-term survival in relapsed ALL. Since 2017, four new immunotherapies have received FDA approval for relapsed B-ALL: blinatumomab, inotuzumab ozogamicin, and two chimeric antigen receptor (CAR) T-cell constructs (tisagenlecleucel and brexucabtagene autoleucel). These agents have drastically improved outcomes for relapsed, adult B-ALL and allow providers to choose a targeted approach based on patient and disease characteristics. Frustratingly, these advances have not yet made strides for patients diagnosed with relapsed T-ALL, with no new FDA approved therapiessince 2005 although active research with early phase clinical trials utilizing immunotherapy based approaches are ongoing.

## Treatment Options for Patients Diagnosed With Relapsed B cell-ALL

### CD19 Targeting

CD19 is essential for the function and maintenance of B-lineage cells, [5] and it is expressed on pre-B cells through their terminal differentiation into plasma cells [6]. Due to its almost universal presence and uniform expression on B-ALL, CD19 serves as one of the disease’s most reliable surface biomarkers and is the target for blinatumomab and the two recently FDA-approved CAR products for relapsed B-ALL [7, 8•, 9•, 10•]. Both blinatumomab and the CD19 CAR therapies function by engaging the cytotoxic potential of T-cells without the need for T-cell receptor specificity, antigen processing/presentation, or major histocompatibility complex context [11, 12].

#### Blinatumomab

Blinatumomab is a bispecific T-cell engager (BiTE) antibody for CD19 and CD3. By binding CD19 on B-cells and CD3 on primarily cytotoxic CD8 + T-cell (CTLs), blinatumomab results in T-cell activation and a cytotoxic T-cell response against CD19-expressing cells [13]. Blinatumomab received accelerated FDA approval in 2014 for Ph- r/r B-ALL based on initial phase II results [11, 14, 15] with full FDA approval for both Ph- and Ph + r/r B-ALL in 2017 following the TOWER and ALCANTARA trials [8•, 14] (Table 1).

**Table 1:** Pivotal trials for blinatumomab, inotuzumab, and CAR T-Cell in Relapsed B-ALL

	Phase	Population	Sample Size	Median Age (range)	Prior HSCT	≥ 2^nd^ Salvage	ORR	MRD^neg^ CR	Median RFS	Post-Treatment HSCT*	Median OS	Unique Toxicity
Blinatumomab												
TOWER^8^	III	R/R Adult (Ph-)	271	41 (18–80)	35%	45%	CR/CRh/CRi: 44%	76%	6.7 mo	14%	7.7 mo	CRS (any): 14% Gr. ≥ 3: 5% ICANS (any): 45% Gr. ≥ 3: 9%
ALCANTARA^14^	II	R/R Adult (Ph +)	45	55 (23–78)	44%	82%	CR/CRh: 36%	88%	6.7 mo	16%	7.1 mo	CRS (any): 7% Gr. ≥ 3: 0% ICANS (any): 47% Gr. ≥ 3: 7%
BLAST^16^	II	MRD + Adult	116	45 (18–76)	0%	26%	NR	78%	18.9 mo	67%	36.5 mo	CRS (any): 3% Gr. ≥ 3: 2% ICANS (any): 53% Gr. ≥ 3: 13%
Inotuzumab												
INO-VATE^66^	III	R/R Adult (Ph ±)	164	46.5 (18–78)	17.7%	34%	CR/CRi = 73.8%	78%	5.0 mo (PFS)	42.7%	7.7 mo	VOD (any): 23% Gr. ≥ 3: 19%
CAR T-Cell												
ELIANA^9^ (Tisa-cel)	II	R/R ≤ 25 yo (Ph ±)	75	11 (3–23)	61%	Median of 3 prior therapies	CR/CRi = 81%	100%	59% at 12 mo	13%	19.1 mo	CRS (any): 77% Gr. ≥ 3: 35% ICANS (any): 40% Gr. ≥ 3: 13%
ZUMA-3^10^ (Brexu-cel)	II	R/R > 18 yo (Ph ±)	55	40 (28–52)	42%	87%	CR/CRi = 71%	97%	14.5 mo	10%	18.2 mo	CRS (any): 93% Gr. ≥ 3: 31% ICANS (any): 78% Gr. ≥ 3: 38%

R/R – relapsed/refractory; Ph – Philadelphia chromosome; HSCT – hematopoietic stem cell transplant; ORR – overall response rate; MRD – minimal residual disease; CR – complete remission; CRh – complete remission with partial hematologic recovery; CRi – complete remission with incomplete hematologic recovery; RFS – relapse free survival; OS – overall survival; CRS – cytokine release syndrome; ICANS—immune effector cell-associated neurotoxicity syndrome; VOD – veno-occlusive disease

^*^ Proceeded to HSCT without receiving additional lines of therapy

The phase III randomized TOWER trial showed higher composite CR rates in patients with r/r Ph- ALL treated with blinatumomab compared to chemotherapy (44% vs 25%), and blinatumomab was more likely to result in a minimal residual disease negative (MRD-neg) CR (76% vs 48%). Median OS was 7.7 months with the greatest benefit for patients in first or second salvage and HSCT-naïve [8•]. The ALCANTARA trial demonstrated a similar CR/CRh rate (36%) with an MRD-neg CR in 88% of responders in patients with relapsed Ph + B-ALL or refractory to tyrosine kinase inhibitors (TKIs) [14].

In 2018, the FDA labeling was expanded to include MRD + B-ALL based on the single arm phase II BLAST trial for adult patients in morphologic remission but detectable MRD > 10^–3^ (Table 1). After one cycle of blinatumomab, 78% of MRD + patients achieved an MRD-neg remission with improved RFS (23.6 mo v 5.7 mo) and OS (38.9 mo v 12.5 mo) when compared to MRD non-responders [16•]. Similar to the TOWER trial, OS was superior in first salvage but there was no long-term survival advantage for patients undergoing consolidative HSCT [17]. Subsequent long-term follow-up of the TOWER trial also showed HSCT may not provide an OS benefit in patients achieving a CR/CRh [18].

The results of the pivotal blinatumomab trials are confirmed in real-world analysis with a CR/CRi rate of 65% in r/r patients (and a 73% MRD-neg) and 75% of the MRD + cohort achieving a remission that is MRD-neg. A third of patients (35.5%) were bridged to HSCT with a 2-year PFS and OS of 66% and 62% respectively [19]. For older patients (ages > 55 years old), efficacy and tolerability were also demonstrated including patients with relapse post prior allogeneic HSCT [20, 21].

#### Toxicity

Due to its unique mechanism of action, blinatumomab has lower rates of grade ≥ 3 neutropenia (37.8% vs 57.8%) and infection (34.1% vs 52.3%), but it can result in a global inflammatory response leading to serious toxicities of cytokine release syndrome (CRS) and immune effector cell-associated neurotoxicity syndrome (ICANS). Across trials, CRS has been reported in 15% of r/r B-ALL patients and 7% of MRD + patients with recommendations for dexamethasone prophylaxis and an initial low-dose infusion period of blinatumomab in order to decrease the incidence of CRS through a slower tumor debulking [22]. ICANS occurs in ~ 65% of all patients receiving blinatumomab but only 13% of events are grade ≥ 3. Rates of severe ICANS appear to be lower in pediatric patients and increased in individuals ≥ 65 years old [20].

Given its short half-life, toxicities like CRS and ICANS can be mitigated quickly with discontinuation of drug infusion and the use of corticosteroids. Compared to salvage chemotherapy approaches, blinatumomab results in a better quality of life and has no increased risk of post-HSCT complications when using blinatumomab as a bridge to transplant [23–25].

#### Limitations of Blinatumomab

A major limitation to blinatumomab efficacy is decreased efficacy in the presence of high disease burden with bone marrow blasts > 50% at time of treatment. Specifically, the TOWER trial showed a decreased CR/CRh (34.4% vs 64.5%) in patients with high vs low disease burden respectively. This finding was confirmed across multiple trials [26]. Although blinatumomab has been shown to have higher response rates in the MRD + setting compared to r/r B-ALL, the 3-year OS for patients with pre-blinatumomab MRD > 1%, 0.1–1%, and < 0.1% has been shown to be 33%, 58% and 86% respectively. Patients going directly to blinatumomab without bridging therapy also had shorter RFS and OS with 3-year OS of 66% in the bridging cohort compared to 16% in patients directly receiving blinatumomab [27]. Additionally, extramedullary involvement at the time of blinatumomab infusion has also been show to predict inferior treatment response, raising concern for its utility in patients with extramedullary disease (EMD). Of particular concern is the presence of CNS disease, which was identified in 29% of post-blinatumomab relapses [28].

Prior treatment with chemotherapy was shown to adversely affect outcomes in the TOWER trial with improved response rates in patients receiving ≤ 2 prior lines of therapy. [29] A pooled analysis found improved CR/CRh (54% vs 41%), median OS (10.4 vs 5.7 months) and median RFS (10.1 v 7.3 months) in patients receiving blinatumomab as first salvage [30]. Additional mechanisms of blinatumomab resistance may include decline and/or complete loss of CD19 [31, 32], increased activity and level of regulatory T cell (Tregs) in the tumor microenvironment [33], and upregulation of PD-L1 [34]. In the case of KMT2A-rearranged ALL, there have been several case reports that illustrate the potential for blinatumomab to induce lineage switch into an aggressive AML [35].

#### CAR-T: Tisagenlecleucel and Brexucabtagene autoleucel

As a form adoptive cell therapy, CARs are genetically engineered T cells expressing a synthetic antigen receptor construct that reprogram the T-cell’s specificity and function. Like blinatumomab, the two FDA approved CARs, tisagenlecleucel (tisa-cel) and brexucabtagene autoleucel (brexu-cel), result in a CTL response through CD19 targeting leading to extensive proliferation of CAR T cells. Following the release of tumor antigens, non–CAR T cells are also recruited producing further antitumor effect through the process of cross priming [12]. Tisa-cel and brexu-cel are both second generation CARs differentiated primarily by their co-stimulatory domain (4-1BB and CD28, respectively) neither of which have demonstrated clear superiority in B cell-ALL [36].

Based on the results of the phase 2 ELIANA trial, tisa-cel is approved for patients < 25 years of age who have received two prior lines of treatment which have failed to establish disease response/control or with post-HSCT relapse (Table 1). Following Phase I results in 60 patients showing a CR rate of 93%, the ELIANA trial of 75 patients resulted in an 81% CR/CRi rate with 95% of responding achieving MRD-negativity by day 28 [9•]. Among evaluable patients, tisa-cel resulted in a 5 year OS and RFS of 55% and 44%, respectively [37]. A recent 3-year update of the ELIANA trial, with a median follow-up of 38.8 months, confirmed these initial findings, and illustrated the potential of a single CAR infusion to be a curative treatment option for heavily pre-treated young adult patients (3-year RFS of 76% in responders) [38••].

Approved for patients > 18 years old with relapsed ALL, brexu-cel was FDA approved in October 2021 following the results of the phase I/II ZUMA-3 trial [10•] (Table 1). Brexu-cel is the same CAR construct as axicabtagene autoleucel previously evaluated in pediatric patients at the National Cancer Institute [39] although it has altered manufacturing in order to decrease early *ex‐vivo* activation and exhaustion of CAR cells by removing leukemic cells from the leukapheresis product [30]. A total of 55/71 enrolled patients were infused on phase II of ZUMA-3 Trial which demonstrated a CR/CRh rate of 71% (and a 97% MRD-negative rate). In this study, the patients were heavily pre-treated with 45% having relapsed post HSCT, 42% of whom had prior exposure to blinatumomab and 22% of whom had prior inotuzumab. With a median follow-up of 16.4 months, the median DOR and RFS following brexu-cel infusion was 12.8 months and 11.6 months, respectively, with 10% of patients proceeding to consolidative HSCT. Of the 55 patients treated on ZUMA-3, 15% were age 65 or older with all 8 patients achieving a CR (compared to 71% in patients < 65 years old) [40]. In the 2-year follow-up, the median OS in responders was 25.4 months versus 5.5 in historical controls [41••]. Compared to tisa-cel, there is stronger support for the use of post-CAR allogeneic HSCT as consolidative therapy for brexu-cel based on experience with CD28 co-stimulatory domains [39, 42–44].

#### Toxicity

Like blinatumomab, the primary toxicity of concern for CD19 CAR constructs is CRS and ICANS. However, unlike blinatumomab’s short half-life and the ability to abort exposure with drug discontinuation, CAR T-cells are a “live” agent with continued exposure that persists in the body. This leads to the potential for increased severity and duration of CAR mediated toxicities (i.e. there is no immediate “kill switch” to stop the ongoing cascade of CRS/ICANS other than steroids/supportive approaches). Rates of grade ≥ 3 CRS and ICANS were 46% and 13%, respectively, with tisa-cel in the Phase 2 ELIANA trial. For brexu-cel, rates of grade ≥ 3 CRS and ICANS were 24% and 25% in the ZUMA-3 study [41••]. While there has been some indication of higher CAR toxicity in older adults treated with CD19 directed therapy for lymphoma, ZUMA-3 did not report differences in CAR toxicity comparing patients ≥ 65 years and < 65 years of age [40].

Aside from the risk of CRS and ICANS, the need for a pre-infusion lymphodepletion regimen can potentially add additional toxicities and patients are also at risk for prolonged cytopenias and infectious complications post-CAR [45, 46].

#### Limitations of CAR-T

The efficacy of CAR-T therapy, like blinatumomab, is limited by the presence of high disease burden prior to lymphodepleting chemotherapy. High leukemia burden pre-CAR is associated with both a decreased overall survival and an increased risk for CAR-mediated toxicities [47–49]. The ZUMA-3 trial found a CR/CRi rate of 42% for patients with bone marrow blasts > 75% compared to a CR/CRi of 80–91% in patients with lower disease burden treated with brexu-cel [30]. A pooled, real-world analysis of tisa-cel showed superior outcomes in patients with undetectable or low burden disease compared to patients with pre-infusion bone marrow blasts ≥ 5%, CNS-3 disease, or non-CNS EMD. The 12-month OS for low burden and undetectable disease was 85% and 95%, respectively, with a similar EFS of 70–72%. In comparison, high disease burden patients had a 12 month OS of 58% and EFS of 31%. Importantly, patients with bone marrow blasts ≥ 5% were also shown to have higher rates of grade ≥ 3 toxicities including CRS, ICANS, and ICU monitoring [50].

Other clinical features associated with impaired response to CD19 targeted CAR therapy include: blinatumomab non-response, prior chemotherapy exposure, and presence of non-CNS EMD. Compared to patients that had a prior response to blinatumomab, nonresponders to blinatumomab had inferior CR rates in response to tisa-cel (65% versus 93%), as well as worse EFS, RFS and OS [49]. Prior chemotherapy exposure was found to portend a worse OS with a hazard ratio (HR) of 1.4, possibly due to the inferior fitness of the T cells collected to manufacture the CAR T product [50, 51]. Like blinatumomab, the presence of EMD at the time of infusion was independently associated with inferior EFS (HR 1.9) [49]. Although CAR therapy appears to be agnostic to cytogenetic risk group with similar outcomes regardless of karyotype [52, 53], the clinical benefit in specific high risk (cytogenetic or mutation based)ALL, such as rearranged KMT2A, mutated *TP53* and hypodiploid ALL is limited [53–56].

In addition to disease specific limitations, the time required [3–6] to engineer an autologous CAR-T product is be a restricting factor, particularly in proliferative leukemic disease. The initial clinical trials had high dropout rates ranging from 18–32% due to time needed for manufacturing juxtaposed with the infectious complications of leukemia and or death due to disease progression before therapy could be delivered [9•, 10•, 47].

### CD22 Targeting

CD22 is present on 90% of B-ALL cells and functions both as an adhesion molecule for other leukocytes and to increase the threshold for antigen/receptor stimulation [57, 58]. Importantly, it remains detected in most cases of CD19 antigen loss following CD19 directed therapy with blinatumomab or CAR T-cell therapy [59, 60]. Unlike CD19, CD22 is rapidly internalized following antibody binding, a feature that is exploited by the antibody–drug conjugate (ADC) Inotuzumab. Inotuzumab is an anti-CD22 monoclonal antibody that is bound to the cytotoxic anti-tumor antibiotic calicheamicin [61]. Following the binding of inotuzumab to the CD22 antigen on B-ALL cells, the complex is internalized, releasing calicheamicin that attaches to the minor grove of the leukemia DNA resulting in DNA cleavage and subsequent apoptosis [62, 63].

#### Inotuzumab

FDA approval for inotuzumab is based on results of the randomized, phase III INO-VATE trial comparing inotuzumab to the standard of care in r/r B-ALL (Table 1). The CR/CRi rate in the intention-to-treat analysis was 80.7% compared to 29.4% with chemotherapy [64•]. Inotuzumab was also more likely to achieve CR regardless of bone marrow blast (BMB) percentage with no statistically significant difference in CR rate between low (BMB < 50%), moderate (BMB 50–90%) or high (BMB > 90%) disease burden. [65] The majority of responses to inotuzumab occurred following cycle 1 (73%) with 78.4% achieving a MRD-negative CR [64•]. With a median follow-up of 29.6 months, the 3-year OS with inotuzumab was 20% versus 6.5% with standard of care therapy. The survival benefit of inotuzumab was in part due to patients being more likely to proceed with HSCT following inotuzumab (48% vs. 22%) [66••]. Patients with MRD-negative CR consolidated with HSCT after inotuzumab had a median OS of 19.2 months compared to 11.1 months in patients that were MRD-positive [67]. These results with single-agent inotuzumab were confirmed in a multicenter real-word analysis of adult patients diagnosed with ALL treated with inotuzumab outside of clinical trials [68].

For older patients (≥ 55 years of age) who were treated on the INO-VATE trial, there was no statistically significant difference in ORR or MRD-negativity rates compared to younger patients. Older patients were more likely to achieve a CRi (43%) compared to a CR (27%), but there was no difference in median DOR (4.7 mo vs 5.4 mo) in either patient population. OS was shorter in the older cohort (5.6 vs 8.6 months; HR 0.610) likely due to these patients being less likely to proceed to HSCT (28% vs 58%) [69]. Like blinatumomab, outcomes with inotuzumab improved when given as first salvage with a CR/CRi rate of 76% compared to 50% when given as a second or later relapse treatment [70, 71]. The delivery of inotuzumab as a short IV infusion in the outpatient setting allows for the ability to administer the therapy in community oncology practices. Importantly, from a quality of life standpoint, patients had lower rate of hospitalization when compared to traditional salvage chemotherapy [72].

#### Toxicity

Aside from an association with increased risk of myelosuppression and neutropenic fever [64•], the most significant toxicity associated with inotuzumab is veno-occlusive disease (VOD). VOD occurred in 13% of patients treated with inotuzumab on the INO-VATE trial compared to 3% in the standard of care arm. Of those with inotuzumab related VOD, 82% were grade III or higher. For those patients receiving a consolidative HSCT, there was an increased rate of VOD if receiving inotuzumab (18.8–22%) compared to standard chemotherapy [73, 74]. For patients ≥ 55 years of age, VOD post-HSCT occurred in 41% of patients compared to 7% in patients 18–29 years of age and 24% in patients 30–54 years of age [69]. Risk of inotuzumab related VOD post-HSCT can been reduced by several approaches: (1) using fractionated dosing, (2) limiting the number of cycles to ≤ 2, (3) avoiding dual alkylator therapy, (4) confirming normal bilirubin pre-HSCT, (5) allowing for a minimum of 4 weeks between inotozumab and HSCT conditioning regimen initiation, and (6) using ursodiol through day + 90 post HSCT [18, 73–76]. For these reasons, a real-world analysis in adults found lower VOD rates post-HSCT that were comparable to historical rates in patients without inotuzumab exposure[68].

#### Limitations of Inotuzumab

Subgroup analysis from the INO-VATE trial found benefit for all patients achieving CR except Ph + B-ALL and patients with < 70% CD22-positivity. Further analysis following the widespread incorporation of TKI therapy for Ph + B-ALL, showed superior median OS in the Ph + patients compared to other cytogenetic abnormalities (NR vs 9.4 mo) [68]. KMT2A-r B-ALL is a high-risk population, which is known to have a lower average CD22 antigen density and the existence of sub-population of blasts that lack CD22 expression [58]. KMT2A-r B-ALL has been shown in multiple studies to be independently associated with inferior OS [68].

Unlike blinatumomab and CAR, high disease burden does not affect outcomes with inotuzumab, but there also appears to be no significant difference in OS for patients with primary refractory disease highlighting the lack of long-term durability of these therapies [68]. There also appears to be evidence that inotuzumab is effective in relapsed patients with non-CNS EMD with CR rate of 55% and 2-year OS of 18% in this challenging to treat population [77]. Despite the ability to use inotuzumab in the setting of high tumor burden or EMD, durability of remissions is short necessitating consolidative HSCT. Patients consolidated with allogeneic HSCT had a 2-year OS of 39% compared to 13% in those not proceeding to HSCT [66••].

## T-ALL

Tremendous progress has been made in the treatment of relapsed adult B-ALL, however, relapsed T-ALL remains a serious challenge. The only FDA approved therapy for relapsed T-ALL remains Nelarabine. This drug was approved in 2005 following demonstration of its efficacy as a single agent in adult relapsed T-ALL with a modest CR rate of 31% and a 1-year OS of only 28% [78]. Recent incorporation of nelarabine in the Children’s Oncology Group’s upfront pediatric T-ALL (including patients up to 31 years of age) resulted in an improved 5-year DFS of 91% but showed no difference in OS [79]. Based on these results, nelarabine is expected to move to the upfront setting in younger adults and fit older adults likely decreasing its utility in the relapsed setting.

Recently, inclusion of the BCL2 inhibitor Venetoclax has shown promise in combination regimens for r/r T-ALL, although these studies have enrolled small numbers of patients. The CR rate for relapsed T-ALL was 60% when venetoclax was added to a traditional chemotherapy backbone, and a phase I trial combing it with the BCL-X_L_ inhibitor navitoclax resulted in a CR rate of 52.6% [80, 81]. However, the movement of these BH3 mimetics (venetoclax/navitoclax) into frontline setting may limit their efficacy in relapsed T-ALL.

Unfortunately, unlike B-ALL, the development of immune targeting approaches in T-ALL remains in early developmental stages. Given the high expression of CD38 on T-ALL, monoclonal antibodies like daratumab and isatuximab, with proven efficacy in multiple myeloma, have been tried with modest results [82, 83]. A trial with a bispecific T-cell–recruiting antibody, similar to blinatumomab, is underway, which also targets CD38 (NCT05038644). Early phase clinical trials testing CD5 and CD7 CAR-T constructs are also looking to overcome the challenge of fratricide caused by the target antigen being expressed both on the T-ALL cells and the CAR-T product [84–86].

## Challenges and Future Directions

### Treatment Selection Between Novel Agents in B-ALL and Growing Upfront Exposure

To date, no randomized head-to-head comparison exists between blinatumomab, inotuzumab and CAR-T in B-ALL, with the only proposed trial (NCT03628053) previously withdrawn. Concerns exist for the interplay of these treatment approaches and the influence of subsequent therapies. Thankfully, real-world analysis has shown comparable efficacy whether patients receive inotuzumab after blinatumomab or the reverse with response rates of 58% vs 52%, respectively [87]. In regards to CAR, neither prior Intozumab exposure nor blinatumomab have been show to effect outcomes, as long as a patient’s leukemia had a prior response and retained CD19 expression [30, 38••, 42, 49].

While some practice preferences have emerged in relapsed B-ALL (CD19 CAR for post-HSCT relapse or HSCT ineligible, inotuzumab for high disease burden or proliferative disease, and blinatumomab for MRD + disease with an available transplant donor), patient and logistical preferences must be taken into account when selecting therapy. Importantly, as blinatumomab, inotuzumab, and CAR-T therapy all are incorporated into the upfront treatment setting, the majority of patients may be exposed to one or more agents earlier in their disease course and this has the potential to decrease future effective therapy in the relapsed setting [88]. Clinical outcomes for patients with B-ALL with non-response or relapsing post-CAR-T, as well as after relapse following sequential treatment with blinatumomab and inotuzumab, remain dismal [89, 90].

### Role of Allogeneic HSCT for Relapsed B-ALL

Previously, the greatest limitation to durable remissions in relapsed, adult B-ALL was the inability to achieve an MRD-negative remission in order to proceed to allogeneic HSCT. However, with the significant improvement in CR rates in patients treated with blinatumomab, inotuzumab, or CAR-T therapy, the question emerges if there is the same necessity for consolidative HSCT. However, based on the available data, most patients still require HSCT for durable remissions regardless of the product used.

While some patients with MRD + B-ALL treated with blinatumomab were able to achieve durable remissions without HSCT, the majority of patients (61%) not undergoing HSCT ultimately relapse [17]. As a single agent, blinatumomab produces superior outcomes for patients when bridging to a consolidative HSCT [18, 91]. In a retrospective analysis of the inotuzumab trials the OS was significantly improved with HSCT (51% vs 22.8%), and remissions achieved with inotuzumab similarly appear to be short lived without a consolidative HSCT [75].

Compared to blinatumomab and inotuzumab, the possibility for durable remission without HSCT exists with CAR T-cell therapy, but it is dependent on the CAR construct and ongoing CAR T cell persistence. The 3-year RFS on the ELIANA trial for patients responding to the tisa-cel but receiving no subsequent therapy was 76% [38••]. Predictive models for post-CAR relapse based on retrospective analyses are being developed with ongoing b-cell aplasia and next generation sequencing (NGS)-MRD as the sole predictive factors identified to date [92•]. The upcoming CAR-CURE trial (NCT05621291) will seek to answer this question prospectively. For CAR products using a CD28 domain, like brexu-cel, HSCT is still felt to be of central importance for meaningful, long-term disease remissions [39, 47].

## Data Availability

No datasets were generated or analysed during the current study.

## References

[1] Brown PA, Shah B, Advani A, Aoun P, Boyer MW, Burke PW, et al. Acute Lymphoblastic Leukemia, Version 2.2021, NCCN Clinical Practice Guidelines in Oncology. J Natl Compr Canc Netw. 2021;19(9):1079–109. 34551384 10.6004/jnccn.2021.0042

[2] Sasaki K, Jabbour E, Short NJ, Jain N, Ravandi F, Pui CH, et al. Acute lymphoblastic leukemia: A population-based study of outcome in the United States based on the surveillance, epidemiology, and end results (SEER) database, 1980–2017. Am J Hematol. 2021;96(6):650–8. 33709456 10.1002/ajh.26156 PMC9517941

[3] Gökbuget N, Dombret H, Ribera JM, Fielding AK, Advani A, Bassan R, et al. International reference analysis of outcomes in adults with B-precursor Ph-negative relapsed/refractory acute lymphoblastic leukemia. Haematologica. 2016;101(12):1524–33. 27587380 10.3324/haematol.2016.144311 PMC5479605

[4] DeAngelo DJ, Jabbour E, Advani A. Recent Advances in Managing Acute Lymphoblastic Leukemia. Am Soc Clin Oncol Educ Book. 2020;40:330–42. 32421447 10.1200/EDBK_280175

[5] Weiland J, Elder A, Forster V, Heidenreich O, Koschmieder S, Vormoor J. CD19: A multifunctional immunological target molecule and its implications for Blineage acute lymphoblastic leukemia. Pediatr Blood Cancer. 2015;62(7):1144–8. 25755168 10.1002/pbc.25462

[6] Wang K, Wei G, Liu D. CD19: a biomarker for B cell development, lymphoma diagnosis and therapy. Exp Hematol Oncol. 2012;1(1):36. 23210908 10.1186/2162-3619-1-36 PMC3520838

[7] Carter RH, Wang Y, Brooks S. Role of CD19 signal transduction in B cell biology. Immunol Res. 2002;26(1–3):45–54. 12403344 10.1385/IR:26:1-3:045

[8] • Kantarjian H, Stein A, Gokbuget N, Fielding AK, Schuh AC, Ribera JM, et al. Blinatumomab versus Chemotherapy for Advanced Acute Lymphoblastic Leukemia. N Engl J Med. 2017;376(9):836–47. In this muti-institutional Phase 3 study, it was shown that treatment with blinatumomab resulted in significantly longer overall survival than chemotherapy among adult patients with relapsed or refractory B cell precursor ALL. 10.1056/NEJMoa1609783 PMC5881572 28249141

[9] • Maude SL, Laetsch TW, Buechner J, Rives S, Boyer M, Bittencourt H, et al. Tisagenlecleucel in Children and Young Adults with B-Cell Lymphoblastic Leukemia. N Engl J Med. 2018;378(5):439–48. In this global Phase 1–2 study of CART therapy, a single infusion of TISA provided durable remission with long term persistence in patients with CD19 positive relapsed or refractory B cell ALL. 10.1056/NEJMoa1709866 PMC5996391 29385370

[10] • Shah BD, Ghobadi A, Oluwole OO, Logan AC, Boissel N, Cassaday RD, et al. KTE-X19 for relapsed or refractory adult B-cell acute lymphoblastic leukaemia: phase 2 results of the single-arm, open-label, multicentre ZUMA-3 study. Lancet. 2021;398(10299):491–502. KTE-X19 showed a high rate of CR or CRi (71%) in adult patients with relapsed or refractory pre B ALL, with median overall survival not reached in responding patients showing that this therapy has potential to confer long term clinical benefit. 10.1016/S0140-6736(21)01222-8 PMC11613962 34097852

[11] Topp MS, Gokbuget N, Stein AS, Zugmaier G, O’Brien S, Bargou RC, et al. Safety and activity of blinatumomab for adult patients with relapsed or refractory B-precursor acute lymphoblastic leukaemia: a multicentre, single-arm, phase 2 study. Lancet Oncol. 2015;16(1):57–66. 25524800 10.1016/S1470-2045(14)71170-2

[12] June CH, Sadelain M. Chimeric antigen receptor therapy. N Engl J Med. 2018;379(1):64–73. 29972754 10.1056/NEJMra1706169 PMC7433347

[13] Nagorsen D, Kufer P, Baeuerle PA, Bargou R. Blinatumomab: a historical perspective. Pharmacol Ther. 2012;136(3):334–42. 22940266 10.1016/j.pharmthera.2012.07.013

[14] Martinelli G, Boissel N, Chevallier P, Ottmann O, Gokbuget N, Topp MS, et al. Complete hematologic and molecular response in adult patients with relapsed/refractory philadelphia chromosome-positive b-precursor acute lymphoblastic leukemia following treatment with blinatumomab: results from a phase ii, single-arm multicenter. Study J Clin Oncol. 2017;35(16):1795–802. 28355115 10.1200/JCO.2016.69.3531

[15] Topp MS, Gokbuget N, Zugmaier G, Klappers P, Stelljes M, Neumann S, et al. Phase II trial of the anti-CD19 bispecific T cell-engager blinatumomab shows hematologic and molecular remissions in patients with relapsed or refractory B-precursor acute lymphoblastic leukemia. J Clin Oncol. 2014;32(36):4134–40. 25385737 10.1200/JCO.2014.56.3247

[16] • Gokbuget N, Dombret H, Bonifacio M, Reichle A, Graux C, Faul C, et al. Blinatumomab for minimal residual disease in adults with B-cell precursor acute lymphoblastic leukemia. Blood. 2018;131(14):1522–31. The results of this open label, single arm study that treated a population of patients with MRD-positive (≥ 10^–3^) B-cell precursor ALL with blinatumomab monotherapy for up to 4 cycles, a majority (78%) achieved a complete MRD response, which was associated with significantly longer RFS (23.6 vs 5.7 months, p=0.002) and OS (38.9 vs 12.5months; p=0.002) compared with MRD nonresponders. 10.1182/blood-2017-08-798322 PMC6027091 29358182

[17] Gokbuget N, Zugmaier G, Dombret H, Stein A, Bonifacio M, Graux C, et al. Curative outcomes following blinatumomab in adults with minimal residual disease B-cell precursor acute lymphoblastic leukemia. Leuk Lymphoma. 2020;61(11):2665–73. 32619115 10.1080/10428194.2020.1780583

[18] Jabbour EJ, Gokbuget N, Kantarjian HM, Thomas X, Larson RA, Yoon SS, et al. Transplantation in adults with relapsed/refractory acute lymphoblastic leukemia who are treated with blinatumomab from a phase 3 study. Cancer. 2019;125(23):4181–92. 31433496 10.1002/cncr.32335

[19] Badar T, Szabo A, Advani A, Wadleigh M, Arslan S, Khan MA, et al. Real-world outcomes of adult B-cell acute lymphocytic leukemia patients treated with blinatumomab. Blood Adv. 2020;4(10):2308–16. 32453836 10.1182/bloodadvances.2019001381 PMC7252553

[20] Kantarjian HM, Stein AS, Bargou RC, Grande Garcia C, Larson RA, Stelljes M, et al. Blinatumomab treatment of older adults with relapsed/refractory B-precursor acute lymphoblastic leukemia: Results from 2 phase 2 studies. Cancer. 2016;122(14):2178–85. 27143254 10.1002/cncr.30031 PMC11752075

[21] Stein AS, Kantarjian H, Gokbuget N, Bargou R, Litzow MR, Rambaldi A, et al. Blinatumomab for Acute Lymphoblastic Leukemia Relapse after Allogeneic Hematopoietic Stem Cell Transplantation. Biol Blood Marrow Transplant. 2019;25(8):1498–504. 31002989 10.1016/j.bbmt.2019.04.010

[22] Halford Z, Coalter C, Gresham V, Brown T. A systematic review of blinatumomab in the treatment of acute lymphoblastic leukemia: engaging an old problem with new solutions. Ann Pharmacother. 2021;55(10):1236–53. 33435716 10.1177/1060028020988411

[23] Salhotra A, Yang D, Mokhtari S, Malki MMA, Ali H, Sandhu KS, et al. Outcomes of allogeneic hematopoietic cell transplantation after salvage therapy with blinatumomab in patients with relapsed/refractory acute lymphoblastic leukemia. Biol Blood Marrow Transplant. 2020;26(6):1084–90. 32035275 10.1016/j.bbmt.2020.01.029 PMC9129096

[24] Badar T, Szabo A, Litzow M, Burkart M, Yurkiewicz I, Dinner S, et al. Multi-institutional study evaluating clinical outcome with allogeneic hematopoietic stem cell transplantation after blinatumomab in patients with B-cell acute lymphoblastic leukemia: real-world data. Bone Marrow Transplant. 2021;56(8):1998–2004. 33824440 10.1038/s41409-021-01279-w

[25] Topp MS, Zimmerman Z, Cannell P, Dombret H, Maertens J, Stein A, et al. Health-related quality of life in adults with relapsed/refractory acute lymphoblastic leukemia treated with blinatumomab. Blood. 2018;131(26):2906–14. 29739753 10.1182/blood-2017-09-804658 PMC6024638

[26] Yu J, Wang W, Huang H. Efficacy and safety of bispecific T-cell engager (BiTE) antibody blinatumomab for the treatment of relapsed/refractory acute lymphoblastic leukemia and non-Hodgkin’s lymphoma: a systemic review and meta-analysis. Hematology. 2019;24(1):199–207. 30479190 10.1080/16078454.2018.1549802

[27] Cabannes-Hamy A, Brissot E, Leguay T, Huguet F, Chevallier P, Hunault M, et al. High tumor burden before blinatumomab has a negative impact on the outcome of adult patients with B-cell precursor acute lymphoblastic leukemia. A real-world study by the GRAALL. Haematologica. 2022;107(9):2072–80. 10.3324/haematol.2021.280078 10.3324/haematol.2021.280078 PMC9425331 35263986

[28] Aldoss I, Otoukesh S, Zhang J, Mokhtari S, Ngo D, Mojtahedzadeh M, et al. Extramedullary disease relapse and progression after blinatumomab therapy for treatment of acute lymphoblastic leukemia. Cancer. 2022;128(3):529–35. 34633671 10.1002/cncr.33967

[29] Dombret H, Topp MS, Schuh AC, Wei AH, Durrant S, Bacon CL, et al. Blinatumomab versus chemotherapy in first salvage or in later salvage for B-cell precursor acute lymphoblastic leukemia. Leuk Lymphoma. 2019;60(9):2214–22. 30947585 10.1080/10428194.2019.1576872

[30] Shah BD, Bishop MR, Oluwole OO, Logan AC, Baer MR, Donnellan WB, et al. KTE-X19 anti-CD19 CAR T-cell therapy in adult relapsed/refractory acute lymphoblastic leukemia: ZUMA-3 phase 1 results. Blood. 2021;138(1):11–22. 33827116 10.1182/blood.2020009098 PMC9999039

[31] Zoghbi A, Zur Stadt U, Winkler B, Müller I, Escherich G. Lineage switch under blinatumomab treatment of relapsed common acute lymphoblastic leukemia without MLL rearrangement. Pediatr Blood Cancer. 2017;64(11). 10.1002/pbc.26594 10.1002/pbc.26594 28453885

[32] Braig F, Brandt A, Goebeler M, Tony HP, Kurze AK, Nollau P, et al. Resistance to anti-CD19/CD3 BiTE in acute lymphoblastic leukemia may be mediated by disrupted CD19 membrane trafficking. Blood. 2017;129(1):100–4. 27784674 10.1182/blood-2016-05-718395

[33] Duell J, Dittrich M, Bedke T, Mueller T, Eisele F, Rosenwald A, et al. Frequency of regulatory T cells determines the outcome of the T-cell-engaging antibody blinatumomab in patients with B-precursor ALL. Leukemia. 2017;31(10):2181–90. 28119525 10.1038/leu.2017.41 PMC5629361

[34] Kohnke T, Krupka C, Tischer J, Knosel T, Subklewe M. Increase of PD-L1 expressing B-precursor ALL cells in a patient resistant to the CD19/CD3-bispecific T cell engager antibody blinatumomab. J Hematol Oncol. 2015;8:111. 26449653 10.1186/s13045-015-0213-6 PMC4599591

[35] Haddox CL, Mangaonkar AA, Chen D, Shi M, He R, Oliveira JL, et al. Blinatumomab-induced lineage switch of B-ALL with t(4:11)(q21;q23) KMT2A/AFF1 into an aggressive AML: pre- and post-switch phenotypic, cytogenetic and molecular analysis. Blood Cancer J. 2017;7(9):e607. 29016570 10.1038/bcj.2017.89 PMC5637108

[36] Cappell KM, Kochenderfer JN. A comparison of chimeric antigen receptors containing CD28 versus 4–1BB costimulatory domains. Nat Rev Clin Oncol. 2021;18(11):715–27. 34230645 10.1038/s41571-021-00530-z

[37] Rives S, Maude SL, Hiramatsu H, Baruchel A, Bader P, Bittencourt H, et al. S112: tisagenlecleucel in pediatric and young adult patients (Pts) With relapsed/refractory (R/R) B-cell acute lymphoblastic leukemia (b-all): final analyses from the eliana study. HemaSphere. 2022;6:13–4. 10.1097/01.HS9.0000843344.19780.98

[38] •• Laetsch TW, Maude SL, Rives S, Hiramatsu H, Bittencourt H, Bader P, et al. Three-Year Update of Tisagenlecleucel in Pediatric and Young Adult Patients With Relapsed/Refractory Acute Lymphoblastic Leukemia in the ELIANA Trial. J Clin Oncol. 2022:JCO2200642. Tisagenlecleucel provided an overall remission rate of 81% as well as median event free survival of 24 months (OS not reached) in pediatric and young adults with R/R B cell ALL. Favorable long-term safety was also demonstrated in this study in these heavily pretreated patients with relapsed refractory B cell ALL.

[39] Shah NN, Lee DW, Yates B, Yuan CM, Shalabi H, Martin S, et al. Long-term follow-up of CD19-CAR T-cell therapy in children and young adults with B-ALL. J Clin Oncol. 2021;39(15):1650–9. 33764809 10.1200/JCO.20.02262 PMC8274806

[40] Shouse G, Danilov AV, Artz A. CAR T-Cell Therapy in the Older Person: Indications and Risks. Curr Oncol Rep. 2022. 10.1007/s11912-022-01272-6 35420395

[41] •• Shah BD, Ghobadi A, Oluwole OO, Logan AC, Boissel N, Cassaday RD, et al. Two-year follow-up of KTE-X19 in patients with relapsed or refractory adult B-cell acute lymphoblastic leukemia in ZUMA-3 and its contextualization with SCHOLAR-3, an external historical control study. J Hematol Oncol. 2022;15(1):170. This study showed the longest follow up of CART therapy (KTE-X19) with meaningful survival benefit in this multicenter study of relapsed refractory patients with B cell ALL at the time of its publication in 2022. 10.1186/s13045-022-01379-0 PMC9734710 36494725

[42] Hay KA, Gauthier J, Hirayama AV, Voutsinas JM, Wu Q, Li D, et al. Factors associated with durable EFS in adult B-cell ALL patients achieving MRD-negative CR after CD19 CAR T-cell therapy. Blood. 2019;133(15):1652–63. 30728140 10.1182/blood-2018-11-883710 PMC6460418

[43] Pan J, Yang JF, Deng BP, Zhao XJ, Zhang X, Lin YH, et al. High efficacy and safety of low-dose CD19-directed CAR-T cell therapy in 51 refractory or relapsed B acute lymphoblastic leukemia patients. Leukemia. 2017;31(12):2587–93. 28490811 10.1038/leu.2017.145

[44] Summers C, Wu QV, Annesley C, Bleakley M, Dahlberg A, Narayanaswamy P, et al. Hematopoietic cell transplantation after CD19 chimeric antigen receptor T Cell-induced acute lymphoblastic lymphoma remission confers a leukemia-free survival advantage. Transplant Cell Ther. 2022;28(1):21–9. 34644605 10.1016/j.jtct.2021.10.003

[45] Jain T, Knezevic A, Pennisi M, Chen Y, Ruiz JD, Purdon TJ, et al. Hematopoietic recovery in patients receiving chimeric antigen receptor T-cell therapy for hematologic malignancies. Blood Adv. 2020;4(15):3776–87. 32780846 10.1182/bloodadvances.2020002509 PMC7422135

[46] Hill JA, Li D, Hay KA, Green ML, Cherian S, Chen X, et al. Infectious complications of CD19-targeted chimeric antigen receptor-modified T-cell immunotherapy. Blood. 2018;131(1):121–30. 29038338 10.1182/blood-2017-07-793760 PMC5755046

[47] Park JH, Riviere I, Gonen M, Wang X, Senechal B, Curran KJ, et al. Long-term follow-up of CD19 CAR therapy in acute lymphoblastic leukemia. N Engl J Med. 2018;378(5):449–59. 29385376 10.1056/NEJMoa1709919 PMC6637939

[48] Maude SL, Frey N, Shaw PA, Aplenc R, Barrett DM, Bunin NJ, et al. Chimeric antigen receptor T cells for sustained remissions in leukemia. N Engl J Med. 2014;371(16):1507–17. 25317870 10.1056/NEJMoa1407222 PMC4267531

[49] Myers RM, Taraseviciute A, Steinberg SM, Lamble AJ, Sheppard J, Yates B, et al. Blinatumomab nonresponse and high-disease burden are associated with inferior outcomes after CD19-CAR for B-ALL. J Clin Oncol. 2022;40(9):932–44. 34767461 10.1200/JCO.21.01405 PMC8937010

[50] Schultz LM, Baggott C, Prabhu S, Pacenta HL, Phillips CL, Rossoff J, et al. Disease burden affects outcomes in pediatric and young adult b-cell lymphoblastic leukemia after commercial tisagenlecleucel: a pediatric real-world chimeric antigen receptor consortium report. J Clin Oncol. 2022;40(9):945–55. 34882493 10.1200/JCO.20.03585 PMC9384925

[51] Das RK, Vernau L, Grupp SA, Barrett DM. Naïve T-cell deficits at diagnosis and after chemotherapy impair cell therapy potential in pediatric cancers. Cancer Discov. 2019;9(4):492–9. 30630850 10.1158/2159-8290.CD-18-1314 PMC6676489

[52] Leahy AB, Devine KJ, Li Y, Liu H, Myers R, DiNofia A, et al. Impact of high-risk cytogenetics on outcomes for children and young adults receiving CD19-directed CAR T-cell therapy. Blood. 2022;139(14):2173–85. 34871373 10.1182/blood.2021012727 PMC8990372

[53] Molina JC, Chinnabhandar V, Fabrizio VA, Kunicki M, Pacenta H, Rossoff J, et al. Standard cytogenetic risk stratification is not predictive of CD19 CAR outcomes and impact of disease burden varies between cytogenetic risk groups. Blood. 2022;140(Supplement 1):10386–8. 10.1182/blood-2022-165951

[54] Lamble AJ, Myers RM, Taraseviciute A, John S, Yates B, Steinberg SM, et al. Preinfusion factors impacting relapse immunophenotype following CD19 CAR T cells. Blood Adv. 2023;7(4):575-85. 10.1182/bloodadvances.2022007423 10.1182/bloodadvances.2022007423 PMC9979750 35482927

[55] Moskop A, Pommert L, Baggott C, Prabhu S, Pacenta HL, Phillips CL, et al. Real-world use of tisagenlecleucel in infant acute lymphoblastic leukemia. Blood Adv. 2022;6(14):4251–5. 35580324 10.1182/bloodadvances.2021006393 PMC9327536

[56] Zhang X, Lu XA, Yang J, Zhang G, Li J, Song L, et al. Efficacy and safety of anti-CD19 CAR T-cell therapy in 110 patients with B-cell acute lymphoblastic leukemia with high-risk features. Blood Adv. 2020;4(10):2325–38. 32453841 10.1182/bloodadvances.2020001466 PMC7252549

[57] Raponi S, De Propris MS, Intoppa S, Milani ML, Vitale A, Elia L, et al. Flow cytometric study of potential target antigens (CD19, CD20, CD22, CD33) for antibody-based immunotherapy in acute lymphoblastic leukemia: analysis of 552 cases. Leuk Lymphoma. 2011;52(6):1098–107. 21348573 10.3109/10428194.2011.559668

[58] Shah NN, Stevenson MS, Yuan CM, Richards K, Delbrook C, Kreitman RJ, et al. Characterization of CD22 expression in acute lymphoblastic leukemia. Pediatr Blood Cancer. 2015;62(6):964–9. 25728039 10.1002/pbc.25410 PMC4405453

[59] Ereno-Orbea J, Sicard T, Cui H, Mazhab-Jafari MT, Benlekbir S, Guarne A, et al. Molecular basis of human CD22 function and therapeutic targeting. Nat Commun. 2017;8(1):764. 28970495 10.1038/s41467-017-00836-6 PMC5624926

[60] Libert D, Yuan CM, Masih KE, Galera P, Salem D, Shalabi H, et al. Serial evaluation of CD19 surface expression in pediatric B-cell malignancies following CD19-targeted therapy. Leukemia. 2020;34(11):3064–9. 32103145 10.1038/s41375-020-0760-x PMC8919057

[61] Lamb YN. inotuzumab ozogamicin: first global approval. Drugs. 2017;77(14):1603–10. 28819740 10.1007/s40265-017-0802-5

[62] DiJoseph JF, Armellino DC, Boghaert ER, Khandke K, Dougher MM, Sridharan L, et al. Antibody-targeted chemotherapy with CMC-544: a CD22-targeted immunoconjugate of calicheamicin for the treatment of B-lymphoid malignancies. Blood. 2004;103(5):1807–14. 14615373 10.1182/blood-2003-07-2466

[63] de Vries JF, Zwaan CM, De Bie M, Voerman JS, den Boer ML, van Dongen JJ, et al. The novel calicheamicin-conjugated CD22 antibody inotuzumab ozogamicin (CMC-544) effectively kills primary pediatric acute lymphoblastic leukemia cells. Leukemia. 2012;26(2):255–64. 21869836 10.1038/leu.2011.206

[64] • Kantarjian HM, DeAngelo DJ, Stelljes M, Martinelli G, Liedtke M, Stock W, et al. Inotuzumab Ozogamicin versus Standard Therapy for Acute Lymphoblastic Leukemia. N Engl J Med. 2016;375(8):740–53. This study demonstrated that R/R patients with B cell ALL treated with Inotuzumab Ozogamicin had a higher rate of CR (as well as increased PFS and OS and eradication of MRD) compared to treatment with standard of care chemotherapy. 10.1056/NEJMoa1509277 PMC5594743 27292104

[65] DeAngelo DJ, Advani AS, Marks DI, Stelljes M, Liedtke M, Stock W, et al. Inotuzumab ozogamicin for relapsed/refractory acute lymphoblastic leukemia: outcomes by disease burden. Blood Cancer J. 2020;10(8):81. 32769965 10.1038/s41408-020-00345-8 PMC7414105

[66] •• Kantarjian HM, DeAngelo DJ, Stelljes M, Liedtke M, Stock W, Gokbuget N, et al. Inotuzumab ozogamicin versus standard of care in relapsed or refractory acute lymphoblastic leukemia: Final report and long-term survival follow-up from the randomized, phase 3 INO-VATE study. Cancer. 2019;125(14):2474–87. This paper reported the long term survival follow up from the Phase 3 randomized study of Inotuzumab versus standard of care for patients with R/R B cell ALL. It demonstrated that Inotuzumab was associated with a higher likelihood of CR and CRi and allowed for more patients to move to HSCT. 10.1002/cncr.32116 PMC6618133 30920645

[67] Jabbour E, Gokbuget N, Advani A, Stelljes M, Stock W, Liedtke M, et al. Impact of minimal residual disease status in patients with relapsed/refractory acute lymphoblastic leukemia treated with inotuzumab ozogamicin in the phase III INO-VATE trial. Leuk Res. 2020;88:106283. 31790983 10.1016/j.leukres.2019.106283

[68] Badar T, Szabo A, Wadleigh M, Liedtke M, Arslan S, Siebenaller C, et al. Real-world outcomes of adult b-cell acute lymphocytic leukemia patients treated with inotuzumab ozogamicin. Clin Lymphoma Myeloma Leuk. 2020;20(8):556-60 e2. 32291234 10.1016/j.clml.2020.03.004

[69] Jabbour EJ, DeAngelo DJ, Stelljes M, Stock W, Liedtke M, Gokbuget N, et al. Efficacy and safety analysis by age cohort of inotuzumab ozogamicin in patients with relapsed or refractory acute lymphoblastic leukemia enrolled in INO-VATE. Cancer. 2018;124(8):1722–32. 29381191 10.1002/cncr.31249

[70] Cassaday RD, Marks DI, DeAngelo DJ, Jabbour EJ, Advani AS, O’Brien S, et al. Impact of number of cycles on outcomes of patients with relapsed or refractory acute lymphoblastic leukaemia treated with inotuzumab ozogamicin. Br J Haematol. 2020;191(3):e77–81. 32805058 10.1111/bjh.17029

[71] Jabbour E, Stelljes M, Advani AS, DeAngelo DJ, Gokbuget N, Marks DI, et al. Impact of salvage treatment phase on inotuzumab ozogamicin treatment for relapsed/refractory acute lymphoblastic leukemia: an update from the INO-VATE final study database. Leuk Lymphoma. 2020;61(8):2012–5. 32378440 10.1080/10428194.2020.1751839

[72] Marks DI, van Oostrum I, Mueller S, Welch V, Vandendries E, Loberiza FR, et al. Burden of hospitalization in acute lymphoblastic leukemia patients treated with Inotuzumab Ozogamicin versus standard chemotherapy treatment. Cancer Med. 2019;8(13):5959–68. 31436395 10.1002/cam4.2480 PMC6792500

[73] Kantarjian HM, DeAngelo DJ, Advani AS, Stelljes M, Kebriaei P, Cassaday RD, et al. Hepatic adverse event profile of inotuzumab ozogamicin in adult patients with relapsed or refractory acute lymphoblastic leukaemia: results from the open-label, randomised, phase 3 INO-VATE study. Lancet Haematol. 2017;4(8):e387–98. 28687420 10.1016/S2352-3026(17)30103-5

[74] Lima MJGD, Kebriaei P, Lanza F, Cho C, Giralt S, Popradi G, et al. A registry-based, observational safety study of inotuzumab ozogamicin (InO) treatment in patients (pts) with B-cell precursor acute lymphoblastic leukemia (ALL) who proceeded to hematopoietic stem cell transplant (HSCT). J Clin Oncol. 2021;39(15_suppl):7017. 10.1200/JCO.2021.39.15_suppl.7017

[75] Marks DI, Kebriaei P, Stelljes M, Gokbuget N, Kantarjian H, Advani AS, et al. Outcomes of allogeneic stem cell transplantation after inotuzumab ozogamicin treatment for relapsed or refractory acute lymphoblastic leukemia. Biol Blood Marrow Transplant. 2019;25(9):1720–9. 31039409 10.1016/j.bbmt.2019.04.020

[76] Mohty M, Malard F, Abecasis M, Aerts E, Alaskar AS, Aljurf M, et al. Prophylactic, preemptive, and curative treatment for sinusoidal obstruction syndrome/veno-occlusive disease in adult patients: a position statement from an international expert group. Bone Marrow Transplant. 2020;55(3):485–95. 31576023 10.1038/s41409-019-0705-z PMC7051913

[77] Kayser S, Sartor C, Luskin MR, Webster J, Giglio F, Panitz N, et al. Outcome of relapsed or refractory acute B-lymphoblastic leukemia patients and BCR-ABL-positive blast cell crisis of B-lymphoid lineage with extramedullary disease receiving inotuzumab ozogamicin. Haematologica. 2022;107(9):2064–71. 35142153 10.3324/haematol.2021.280433 PMC9425305

[78] DeAngelo DJ, Yu D, Johnson JL, Coutre SE, Stone RM, Stopeck AT, et al. Nelarabine induces complete remissions in adults with relapsed or refractory T-lineage acute lymphoblastic leukemia or lymphoblastic lymphoma: Cancer and Leukemia Group B study 19801. Blood. 2007;109(12):5136–42. 17344466 10.1182/blood-2006-11-056754 PMC1941786

[79] Dunsmore KP, Winter SS, Devidas M, Wood BL, Esiashvili N, Chen Z, et al. Children’s oncology group AALL0434: a phase iii randomized clinical trial testing nelarabine in newly diagnosed T-Cell acute lymphoblastic leukemia. J Clin Oncol. 2020;38(28):3282–93. 32813610 10.1200/JCO.20.00256 PMC7526719

[80] Pullarkat VA, Lacayo NJ, Jabbour E, Rubnitz JE, Bajel A, Laetsch TW, et al. Venetoclax and navitoclax in combination with chemotherapy in patients with relapsed or refractory acute lymphoblastic leukemia and lymphoblastic lymphoma. Cancer Discov. 2021;11(6):1440–53. 33593877 10.1158/2159-8290.CD-20-1465 PMC9533326

[81] Richard-Carpentier G, Jabbour E, Short NJ, Rausch CR, Savoy JM, Bose P, et al. Clinical experience with venetoclax combined with chemotherapy for relapsed or refractory T-Cell acute lymphoblastic leukemia. Clin Lymphoma Myeloma Leuk. 2020;20(4):212–8. 32035785 10.1016/j.clml.2019.09.608

[82] Boissel N, Chevallier P, Doronin V, Griskevicius L, Maschan A, McCloskey J, et al. Isatuximab monotherapy in patients with refractory T-acute lymphoblastic leukemia or T-lymphoblastic lymphoma: Phase 2 study. Cancer Med. 2022;11(5):1292–8. 35106962 10.1002/cam4.4478 PMC8894690

[83] Hogan LE, Bhatla T, Teachey DT, Sirvent FJB, Moppett J, Puyó PV, et al. Efficacy and safety of daratumumab (DARA) in pediatric and young adult patients (pts) with relapsed/refractory T-cell acute lymphoblastic leukemia (ALL) or lymphoblastic lymphoma (LL): Results from the phase 2 DELPHINUS study. J Clin Oncol. 2022;40(16_suppl):10001. 10.1200/JCO.2022.40.16_suppl.10001

[84] Hill LC, Rouce RH, Smith TS, Yang L, Srinivasan M, Zhang H, et al. Safety and anti-tumor activity of CD5 CAR T-Cells in patients with relapsed/refractory T-Cell malignancies. Blood. 2019;134(Supplement_1):199.31064751 10.1182/blood-2019-129559

[85] Pan J, Tan Y, Wang G, Deng B, Ling Z, Song W, et al. Donor-derived CD7 chimeric antigen receptor T Cells for T-Cell acute lymphoblastic leukemia: first-in-human. Phase I Trial J Clin Oncol. 2021;39(30):3340–51. 34324392 10.1200/JCO.21.00389

[86] Li S, Wang X, Yuan Z, Liu L, Luo L, Li Y, et al. Eradication of T-ALL cells by CD7-targeted Universal CAR-T Cells and initial test of ruxolitinib-based CRS management. Clin Cancer Res. 2021;27(5):1242–6. 33234511 10.1158/1078-0432.CCR-20-1271

[87] Badar T, Szabo A, Dinner S, Liedtke M, Burkart M, Shallis RM, et al. Sequencing of novel agents in relapsed/refractory B-cell acute lymphoblastic leukemia: Blinatumomab and inotuzumab ozogamicin may have comparable efficacy as first or second novel agent therapy in relapsed/refractory acute lymphoblastic leukemia. Cancer. 2021;127(7):1039–48. 33259056 10.1002/cncr.33340

[88] Advani A. When will chemotherapy be replaced in upfront induction therapy for adult acute lymphoblastic leukemia (ALL)? Best Pract Res Clin Haematol. 2022;35(4):101404. 36517121 10.1016/j.beha.2022.101404

[89] Schultz LM, Eaton A, Baggott C, Rossoff J, Prabhu S, Keating AK, et al. Outcomes After Nonresponse and Relapse Post-Tisagenlecleucel in Children, Adolescents, and Young Adults With B-Cell Acute Lymphoblastic Leukemia. J Clin Oncol. 2023;41(2):354–63. 36108252 10.1200/JCO.22.01076 PMC9839307

[90] Wudhikarn K, King AC, Geyer MB, Roshal M, Bernal Y, Gyurkocza B, et al. Outcomes of relapsed B-cell acute lymphoblastic leukemia after sequential treatment with blinatumomab and inotuzumab. Blood Adv. 2022;6(5):1432–43. 35042232 10.1182/bloodadvances.2021005978 PMC8905691

[91] Topp MS, Gokbuget N, Zugmaier G, Stein AS, Dombret H, Chen Y, et al. Long-term survival of patients with relapsed/refractory acute lymphoblastic leukemia treated with blinatumomab. Cancer. 2021;127(4):554–9. 33141929 10.1002/cncr.33298 PMC7894150

[92] • Pulsipher MA, Han X, Maude SL, Laetsch TW, Qayed M, Rives S, et al. Next-Generation Sequencing of Minimal Residual Disease for Predicting Relapse after Tisagenlecleucel in Children and Young Adults with Acute Lymphoblastic Leukemia. Blood Cancer Discov. 2022;3(1):66–81. This is one of the first papers to show utility of the use of NGS to evaluate for MRD in predicting relapse after CART therapy in children and young adults diagnosed with ALL. 10.1158/2643-3230.BCD-21-0095 PMC9924295 35019853

